# Supplementary material for: TDP-43 nuclear loss in FTD/ALS causes widespread alternative polyadenylation changes
Source: bioRxiv. 2024 Jan 22:2024.01.22.575730. Preprint. [Version 1] doi: 10.1101/2024.01.22.575730 (PMC10849503; doi:10.1101/2024.01.22.575730)
Supplement: Supplement 8 [file NIHPP2024.01.22.575730v1-supplement-8.pdf]

bioRxiv preprint doi: https://doi.org/10.1101/2024.01.22.575730; this version posted January 22, 2024. The copyright holder for this preprint (which was not certified by peer review) is the author/funder, who has granted bioRxiv a license to display the preprint in perpetuity. It is made available under aCC-BY 4.0 International license.

Figure S1. Loss of TDP-43 leads to widespread alternative polyadenylation changes.

a, Volcano plot shows that QAPA, another popular APA analysis program, also uncovered that loss of TDP-43 is associated with APA changes. The log2(fold change) represents the difference of distal polyA site usage between TDP-43 negative nuclei and TDP-43 positive nuclei. Adjusted p-values were calculated by DEXSeq. **b**, Volcano plot shows that genes have both APA changes and RNA level changes. Only genes with significant APA changes, detected by APAlyzer or QAPA, are plotted with RNA level change on the x-axis and the corresponding adjusted p value on the y-axis. Differential RNA expression was analyzed using DESeq2. **c-e**, Volcano plots show that APAlyzer-identified APA changes upon TPD-43 knockdown (KD) in iMNs (**c**), in iNeurons carrying K263E mutation in TDP-43 (**d**), and in iMNs carrying M337V mutation in TDP-43 (**e**). Details, as in Fig. 1e.

bioRxiv preprint doi: https://doi.org/10.1101/2024.01.22.575730; this version posted January 22, 2024. The copyright holder for this preprint (which was not certified by peer review) is the author/funder, who has granted bioRxiv a license to display the preprint in perpetuity. It is made available under aCC-BY 4.0 International license.

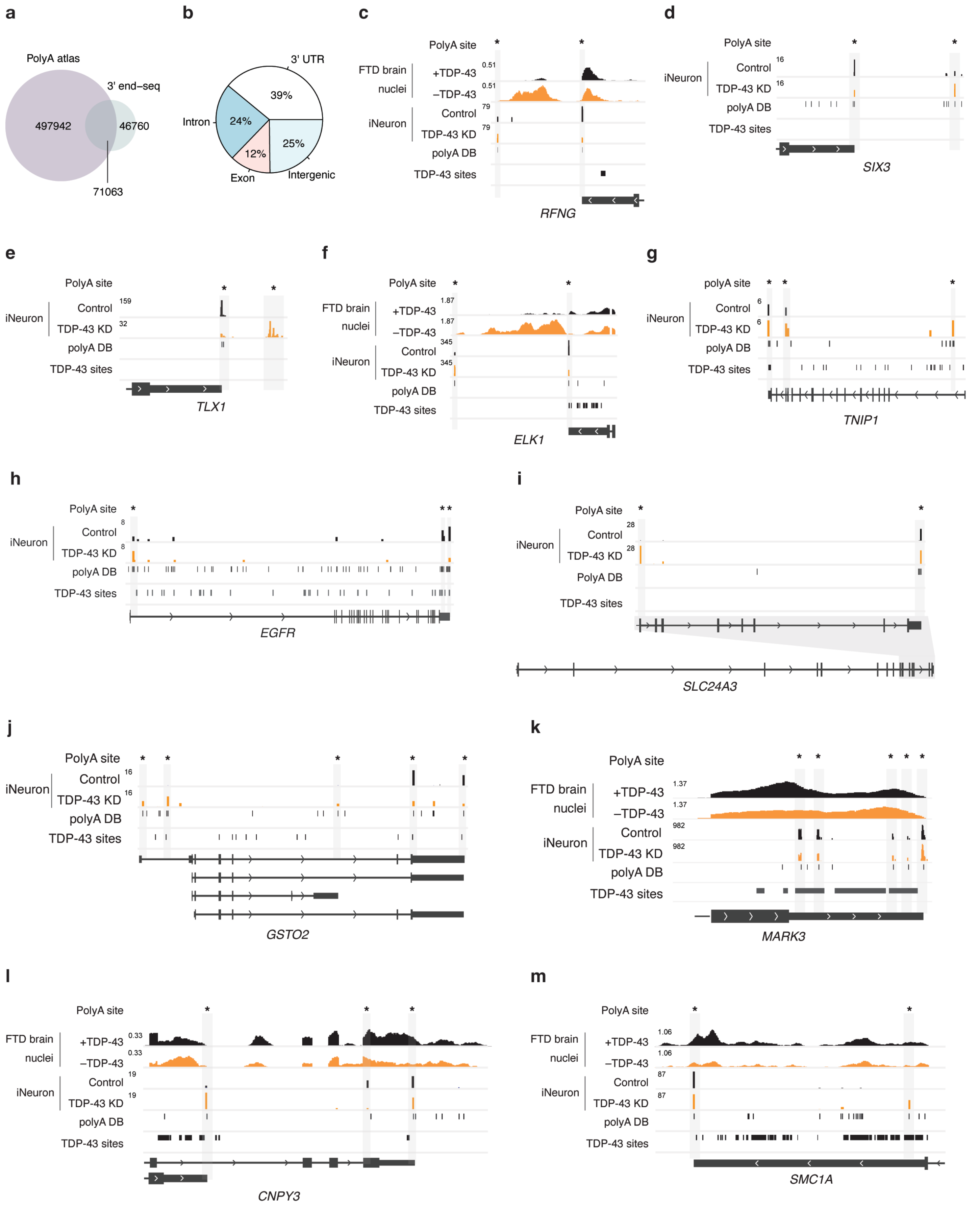

Figure S2. High-resolution mapping of TDP-43 dependent alternative polyadenylation using 3' end-seq.

bioRxiv preprint doi: https://doi.org/10.1101/2024.01.22.575730; this version posted January 22, 2024. The copyright holder for this preprint (which was not certified by peer review) is the author/funder, who has granted bioRxiv a license to display the preprint in perpetuity. It is made available under aCC-BY 4.0 International license.

a, Venn Diagram illustrates that 3' end-seq captured annotated and novel polyA sites. Reads from both control and TDP-43 knockdown (KD) samples were used. **b**, Pie chart shows the distribution of 3' end-seq identified polyA sites. Note that "intergenic" represents polyA sites not associated with annotated 3' UTRs, exons, or introns. **c-f**, Examples of the use of an unannotated distal polyA site upon TDP-43 KD. **g-j**, Examples of premature polyadenylation upon TDP-43 KD. **k**, Example of complex usage change of multiple polyA sites upon TDP-43 KD. **l**. Example of increased proximal polyA usage upon TDP-43 KD. **m**, Example of 3' UTR shortening upon TDP-43 KD. Details, as in Fig. 2g.

bioRxiv preprint doi: https://doi.org/10.1101/2024.01.22.575730; this version posted January 22, 2024. The copyright holder for this preprint (which was not certified by peer review) is the author/funder, who has granted bioRxiv a license to display the preprint in perpetuity. It is made available under aCC-BY 4.0 International license.

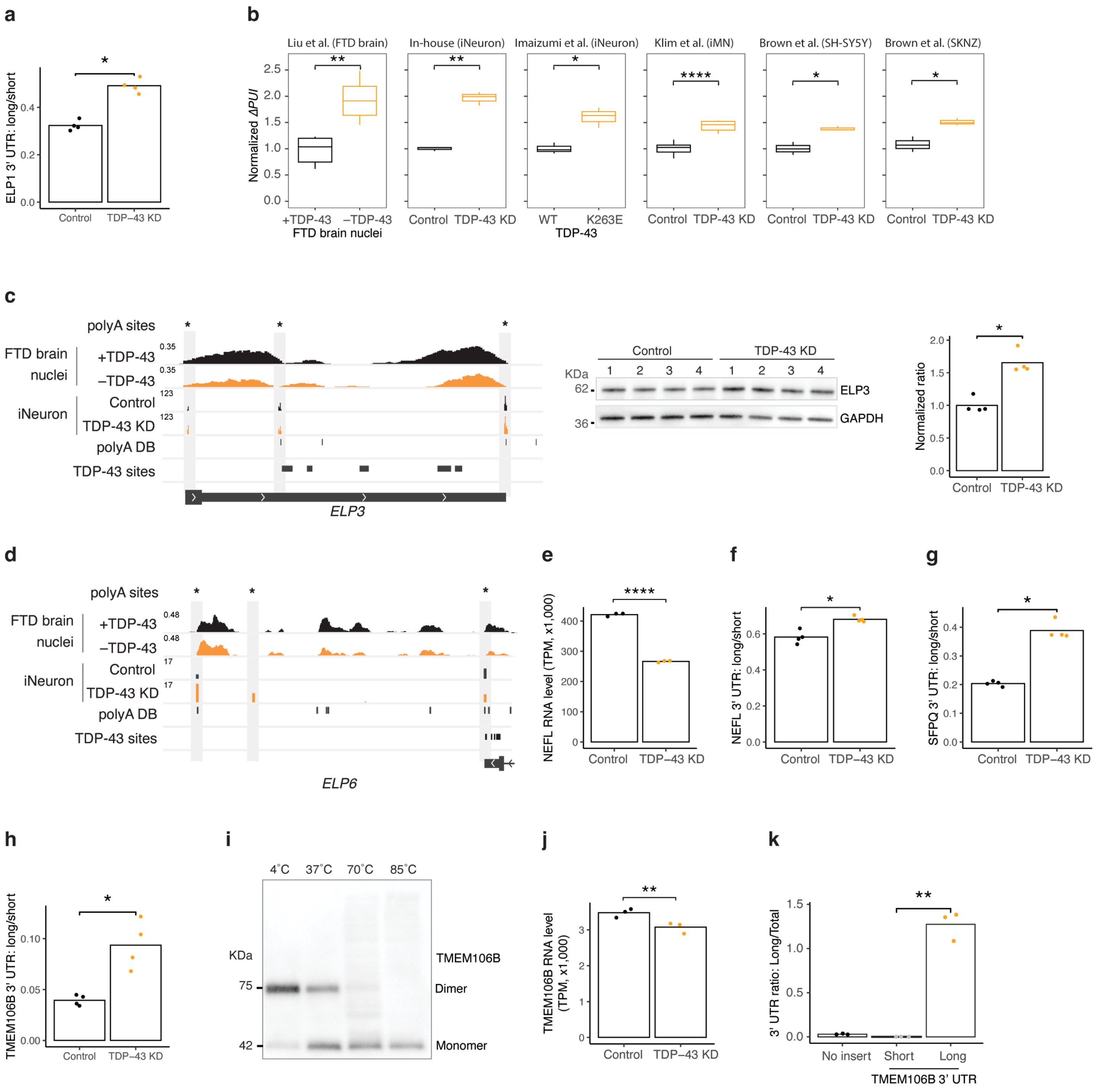

Figure S3. Loss of TDP-43 causes alternative polyadenylation that alters protein levels of disease-associated genes.

a, Bar plots show that TDP-43 knockdown (KD) increased the level of the long *ELP1* 3' UTR, confirmed by qRT-PCR. **b**, Bar plots show that higher level of the long *ELP1* 3' UTR is present across diverse datasets in which TDP-43 was either knocked down or carrying a pathogenic mutation. Normalized Δ*PUI* was calculated as followed: normalized read coverage in the long 3' UTR region was divided by normalized read coverage in the common 3' UTR region and the resulting ratio was normalized to the condition that TDP-43 was functional. **c**, TDP-43 KD caused complex APA changes in the 3' UTR of *ELP3* (**left panel**), increasing its protein levels (**middle and right panels**). **d**, TDP-43 KD lengthened the 3' UTR of *ELP6*. **e**, Bar plots show that TDP-43 KD reduced *NEFL* RNA levels. The adjusted *p* value was calculated in DEseq2. **f-h**, Bar plots show that TDP-43 KD increased levels of long 3' UTRs of *NEFL* (**f**), *SFPQ* (**g**), and

bioRxiv preprint doi: https://doi.org/10.1101/2024.01.22.575730; this version posted January 22, 2024. The copyright holder for this preprint (which was not certified by peer review) is the author/funder, who has granted bioRxiv a license to display the preprint in perpetuity. It is made available under aCC-BY 4.0 International license.

TMEM106B (**h**), confirmed by qRT-PCR. **i**, WB shows that as the temperature increases, a ~75 KDa band collapses to a 42 KDa band. Cell lysates were incubated at respective temperatures for 10 min before electrophoresis. **j**, Bar plots show that TDP-43 KD modestly reduced *TMEM106B* RNA levels. The adjusted *p* value was calculated in DEseq2. **k**, Bar plots show that the reporter with the long *TMEM106B* 3' UTR produced the long 3' UTR, as designed. RNA levels were measured by qRT-PCR. Unless stated otherwise, *p*-values were calculated by Student's *t* test. ns (not significant), $p > 0.05$; *, $p \leq 0.05$; **, $p \leq 0.01$; ***, $p \leq 0.001$; ****, $p \leq 0.0001$.

bioRxiv preprint doi: https://doi.org/10.1101/2024.01.22.575730; this version posted January 22, 2024. The copyright holder for this preprint (which was not certified by peer review) is the author/funder, who has granted bioRxiv a license to display the preprint in perpetuity. It is made available under aCC-BY 4.0 International license.

List of supplementary tables

Table S1: Alternative polyadenylation changes detected in Liu et al. 2019

Table S2: Significant APA changes in 3' UTRs upon TDP-43 knockdown detected by RNA-seq

Table S3: Significant APA changes upon TDP-43 knockdown detected by 3' end-seq

Table S4: Curated list of FTD and ALS related genes

Table S5: Cryptic polyA sites activated by TDP-43 knockdown

Table S6: List of primers used in qRT-PCR

Table S7: Summary of patient data
